# Verrucous Melanoma of the Scalp Initially Misdiagnosed as Seborrheic Keratosis

**DOI:** 10.7759/cureus.29098

**Published:** 2022-09-13

**Authors:** Amal Kerouach, Fouzia Hali, Sarah Belanouane, Farida Marnissi, Soumiya Chiheb

**Affiliations:** 1 Dermatology, Ibn Rochd University Hospital Center, Casablanca, MAR; 2 Anatomical Pathology, Ibn Rochd University Hospital Center, Casablanca, MAR

**Keywords:** melanocytic lesion, melanoma, hyperkeratotic melanoma, seborrheic keratosis, verrucous melanoma

## Abstract

Verrucous melanoma (VM) is a rare entity that presents diagnostic difficulty on both clinical and histopathologic grounds. Clinically, this tumor can be mistaken for a benign non-melanocytic lesion, particularly seborrheic keratosis (SK), as they both share several similarities, such as the homogenous pigmentation, the verrucous surface, and the roughly well-defined borders. In our patient’s case, her verrucous lesion was initially misdiagnosed as SK by a general practitioner two months prior to her admission. Upon physical examination, the lesion was indeed suggestive of SK but a VM was not discarded. Biopsy revealed melanoma. Standard treatment of SK often includes electrodesiccation or cryotherapy, which potentially might worsen and delay the diagnosis of melanoma with subsequent implications for therapeutic management and prognosis. We report this case to increase awareness and knowledge about VM, which may lead to earlier diagnosis and improved outcomes.

## Introduction

Verrucous melanoma (VM) is an extremely rare variant of melanoma that is still challenging to diagnose and differentiate from other benign verrucous lesions, particularly seborrheic keratosis (SK) [1-3]. This case highlights the importance of recognizing the similarities and differences between the two conditions as treatment of SK often includes techniques that can potentially worsen and delay the diagnosis of melanoma.

## Case presentation

**Figure 1:**
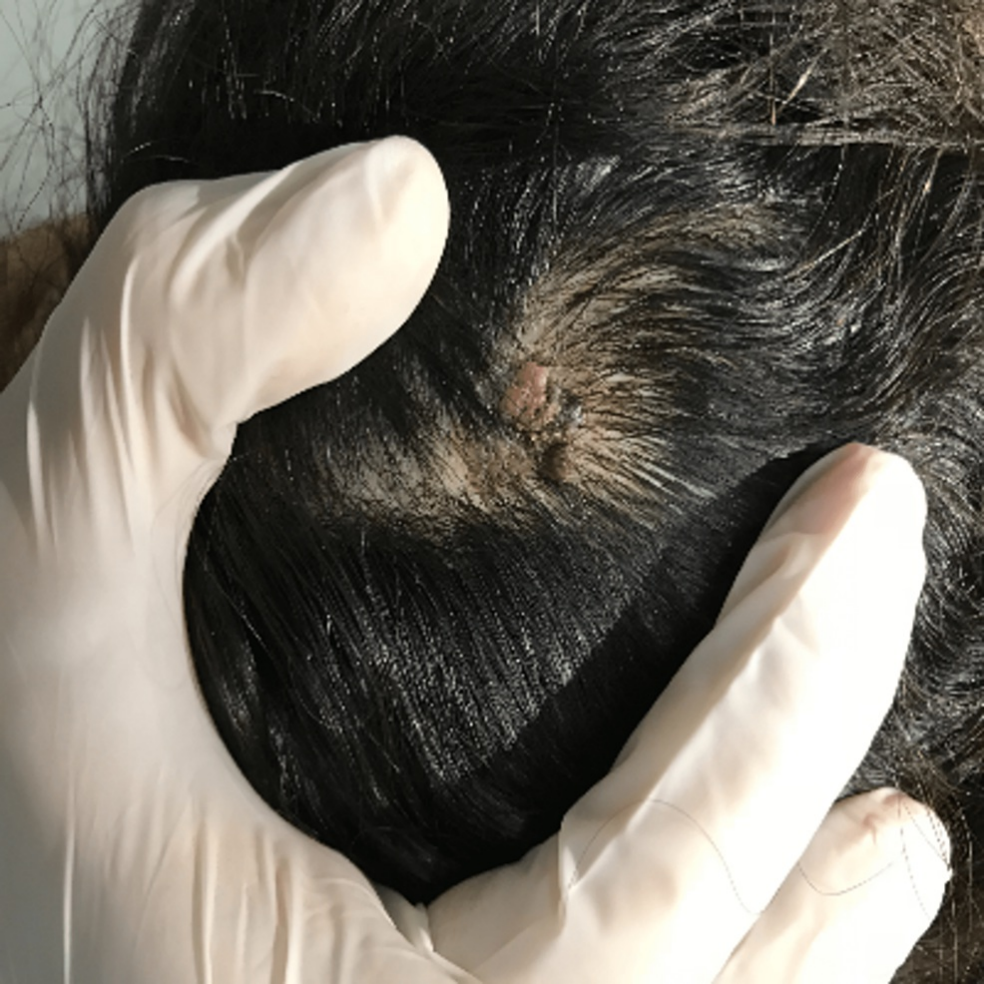
Verrucous lesions upon physical examination at the time of admission.

**Figure 2:**
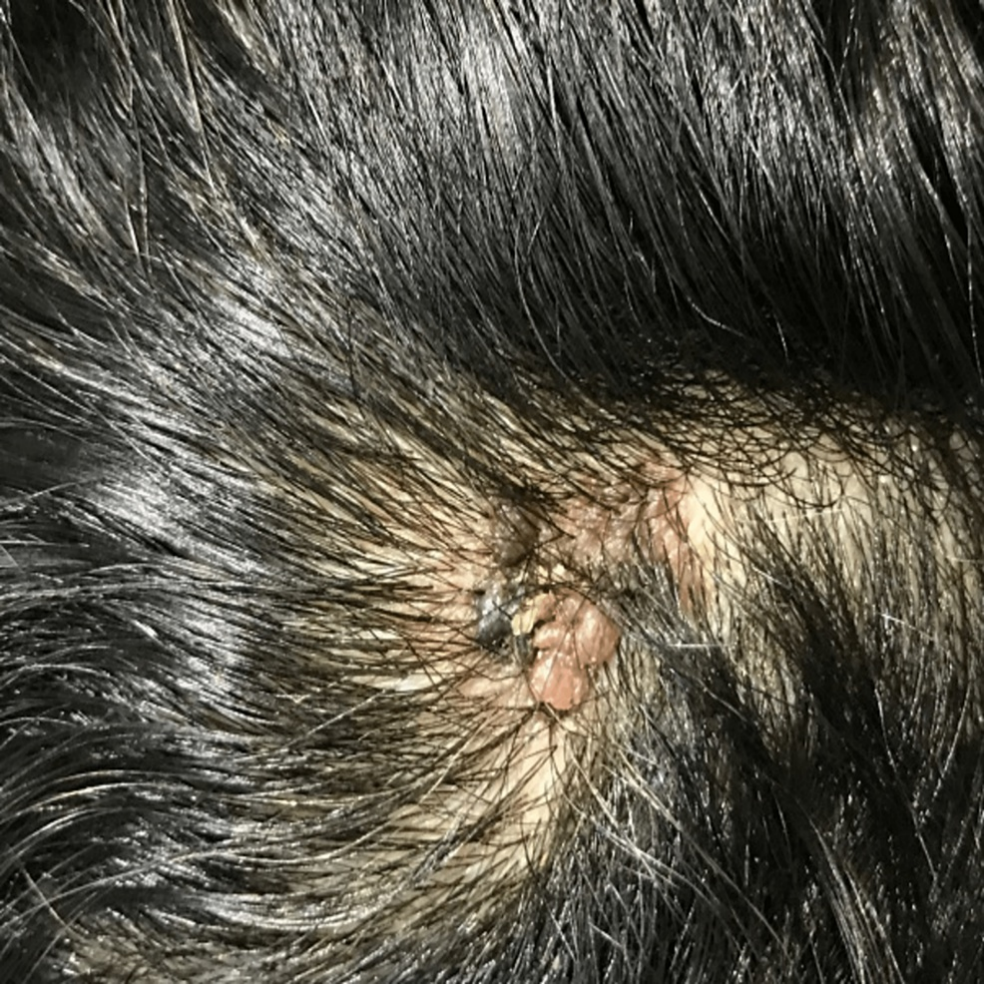
Verrucous melanoma: a close-up view of the scalp area shows a hyperpigmented lesion with abrupt borders and verrucous surface and keratin plugs.

**Figure 3:**
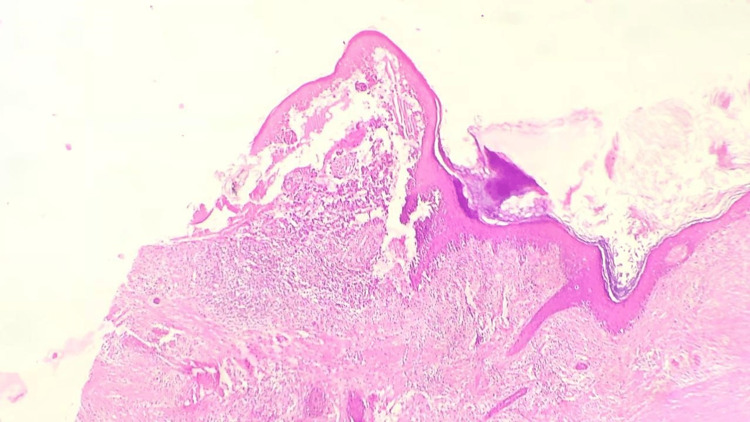
Verrucous melanoma: biopsy showing papilliferous acanthosis and hyperplasia of the epidermis with elongated rete ridges with orthohyperkeratosis.

**Figure 4:**
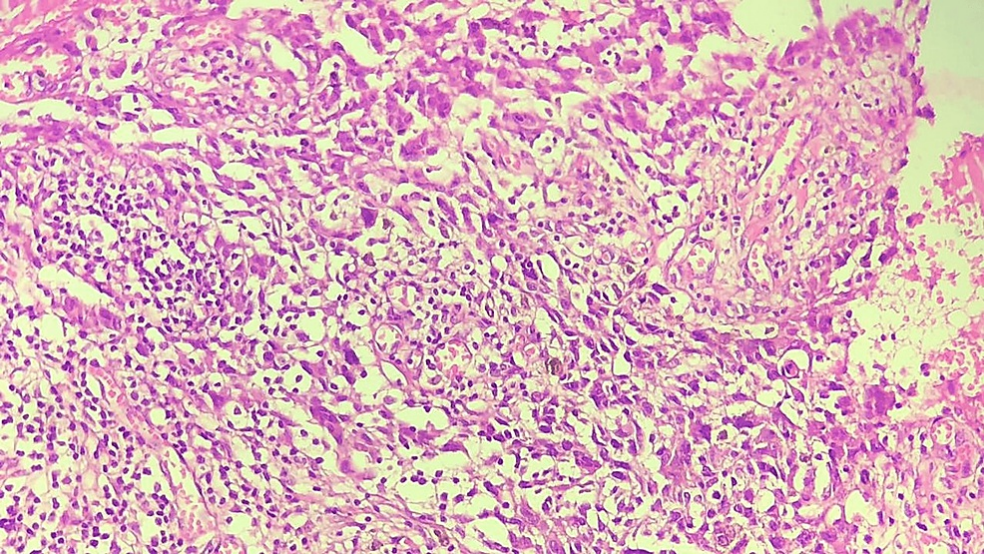
Magnification of the biopsy shows features of an invasive melanoma with verrucous features extending to the base and peripheral biopsy edges (hematoxylin and eosin ×20).

**Figure 5:**
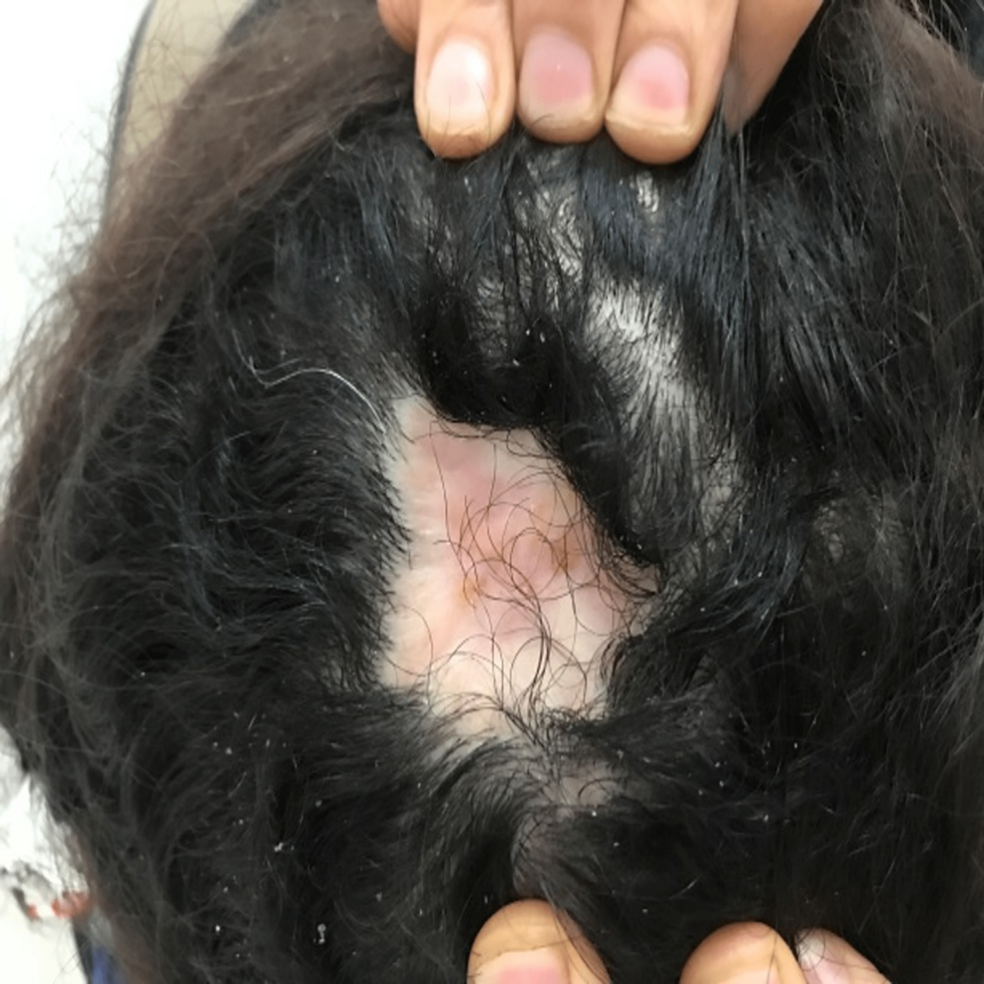
Verrucous melanoma at two months after excision.

A 46-year-old woman presented to our outpatient dermatology department complaining of a painless, verrucous lesion of the scalp that had been present for four months (Figure 1). Upon clinical examination, we noted an oval brownish-black hyperkeratotic lesion with irregular raised borders and a verrucous surface covered with a squamous, keratotic film that was not friable at curettage (Figure 2). During the four-month evolution, the lesion had gradually enlarged but no associated signs (bleeding, pain, etc.) were reported or found. A prior diagnosis of SK had been given by a general practitioner. At our practice, VM was suspected and included in the differential diagnosis. A biopsy was performed, and the findings were consistent with papilliferous acanthosis and hyperplasia of the epidermis with elongated rete ridges with orthohyperkeratosis, anisokaryosis, and atypical fragmented melanocytes mostly arranged in nests along the dermis (Figure 3). Melanocytes were positive for S-100 protein, HMB45, and Melan-A. The Ki-67 proliferation index was hard to estimate due to the important epidermal hyperplasia but seemed minimal (Figure 4). The entire lesion was then removed with 2 cm lateral margins and subjected to histopathological investigation, which revealed a superficially spreading melanoma with a Breslow depth of 2 mm and Clark Level III. No signs of vascular or neurological involvement were noted on the biopsy. Brisk tumor-infiltrating lymphocytes were present throughout the tumoral growth. The positron emission tomography did not show metastases. The patient is currently under regular surveillance with a 26-month follow-up (Figure 5).

## Discussion

VM is an extremely rare melanoma that may develop on any conventional clinical type of melanoma or arise de novo as a primary tumor. When it occurs de novo, it is believed that VM has a better prognosis than when it is secondary [1-3]. A retrospective study conducted in Argentina studied all melanoma cases hospitalized between January 1991 and September 2007 [1]. Among the 545 melanoma cases, only four (0.7%) cases were primary or de novo VM, and six (0.9%) were secondary VM, emphasizing the rarity of this entity.

Overall, 71% of cases of this melanoma type are located on the upper and lower extremities [3]. It equally affects males and females and has a similar prognosis, with cure rates ranging between 95% and 100% [1]. VM is considered a clinical variant rather than a clinical entity in the 2000 Australian guidelines [4].

SK are benign non-melanocytic tumors that are well-known to dermatologists due to their characteristic clinical features, and it is a common and accepted practice to eliminate them without histopathologic confirmation. However, recent reports showed that several cases of VM were initially misdiagnosed as SK [1-4], even more, it was reported that malignant melanoma could arise within SK [5].

In 1967, Clark initially classified melanoma into four clinical types: lento-maligna melanoma (LMM), superficial spreading melanoma (SSM), nodular melanoma (NM), and VM. Later, Clark et al. eliminated VM from their classification and included acral lentiginous melanoma (ALM), arguing that any type of melanoma, especially SSM, could develop a verrucous consistency. Consequently, he dropped VM from the classification, which caused this type of melanoma to become less known and reported in the literature and was no longer individualized as an entity and not often included in the differential diagnoses of similar benign lesions [1,2], except melanoacathoma.

Clinically, it may be challenging to differentiate between VM and SK. Both share many similarities, such as homogenous pigmentation, the verrucous surface, and roughly well-defined borders [1]. Despite the resemblance, consistency might be an important feature to help distinguish these two entities. The consistency of VM is fleshier in contrast with the friable texture of SK. We suggest that curetting such benign-looking lesions upon examination might help suspect the diagnosis of VM if the consistency proves to be fleshy instead of friable, as expected with SK. An incisional biopsy is highly recommended.

This case highlights the clinical existence of such benign-looking melanomas. Steiner et al. reported five cases of VM that were initially clinically diagnosed as SK [2]. In their report, they described the clinical characteristics of VM as follows: (1) hyperkeratotic verrucous surface with keratotic plugs and some scaling; (2) relatively uniform dark pigmentation with a grayish aspect; (3) sharp demarcation and moderately irregular outline; and (4) tough and fleshy consistency. All these characteristics were found in our patient.

Such benign-looking melanomas usually lack the validity of the ABCD rule; therefore, Giacomo et al. proposed the additional clinical features known as the EFG rule referring to elevated, firm, and growing types of melanomas [6].

Dermoscopic inspection of VM is also challenging as it shows, in addition to the classic melanocytic patterns such as dots, globules, streaks, and a bluish-white veil, the presence of comedo-like openings and milia-like cysts, also commonly found in SK [7]. Although dermoscopic features are characteristic, it is safe to say that relying on clinical and dermatoscopic diagnosis without histologic analysis to differentiate the two lesions turns out to be imprudent, as shown by multiple studies [8,9]. We can easily deduct that only histologic analysis approaches 100% reliability [10].

As shown in our case, and according to the literature, histologic findings in most of the reported cases were in keeping with SSM or ALM [1,2,11], the histopathologic features are mainly epidermal hyperplasia with elongated rete ridges. Orthohyperkeratosis, irregular parakeratosis or both, fragmented atypical melanocytes scattered throughout the basal layer and through the epidermis, and distinctively a discordance between Breslow depth and Clark’s level, which was found in almost all of the reported cases, consisting of a clinically advanced looking lesion contrasting with a moderate tumor thickness that can be explained by the important epidermal hyperplasia and hyperkeratosis.

VM has a more favorable prognosis [1,2]. Our patient was followed up for 26 months without signs of relapse. A recent meta-analysis confirmed the favorable prognostic role of the TILs in the overall survival of melanoma patients and found an association between the presence of TILs and improved survival. Our histology showed a very important brisk TIL infiltration [12].

Electrodesiccation and carbon dioxide laser removal of SK are frequent in our practice. Such procedures if used on melanoma might worsen the diagnosis. Therefore, it is important for pathologists to recognize this unusual variant to adapt the treatment.

## Conclusions

SK is a benign condition that can mimic a large range of neoplasms, both melanoma and non-melanoma. Excluding VM in the differential diagnosis of SK might lead to misdiagnosis, with subsequent implications for therapeutic management and prognosis. We report the present case with the aim of increasing awareness and knowledge about VM, which may lead to earlier diagnosis and improved outcomes.
